# Rationale and Protocol of the Multimodality Evaluation of Antibody-Mediated Injury in Heart Transplantation (LEONE-HT) Observational Cross-Sectional Study

**DOI:** 10.3390/mps5050075

**Published:** 2022-09-25

**Authors:** Jorge Nuche, Javier de la Cruz Bertolo, Irene Marco Clement, Violeta Sánchez Sánchez, Fernando Sarnago Cebada, Esther Mancebo, Ana Belén Enguita, Marina Alonso-Riaño, Gema Ruiz-Hurtado, Juan Carlos López-Azor, Francisco José Hernández-Pérez, Javier Castrodeza, Javier Sánchez González, Fernando Arribas Ynsaurriaga, María Dolores García-Cosío Carmena, Juan F. Delgado

**Affiliations:** 1 Cardiology Department, Hospital Universitario 12 de Octubre, Instituto de Investigación Sanitaria Hospital 12 de Octubre (imas12), Avenida de Andalucía s/n, 28041 Madrid, Spain; 2 Centro de Investigaciones Biomédicas En Red de enfermedades CardioVasculares (CIBERCV), 28029 Madrid, Spain; 3 Centro Nacional de Investigaciones Cardiovasculares, Calle Melchor Fernández Almagro 3, 28029 Madrid, Spain; 4 Epidemiology and HS/TA, Hospital Universitario 12 de Octubre, Instituto de Investigación Sanitaria Hospital 12 de Octubre (imas12), Avenida de Andalucía s/n, 28041 Madrid, Spain; 5 Immunology Department, Hospital Universitario 12 de Octubre, Instituto de Investigación Sanitaria Hospital 12 de Octubre (imas12), Avenida de Andalucía s/n, 28041 Madrid, Spain; 6 Pathology Department, Hospital Universitario 12 de Octubre, Instituto de Investigación Sanitaria Hospital 12 de Octubre (imas12), Avenida de Andalucía s/n, 28041 Madrid, Spain; 7 Cardiorenal Translational Laboratory, Instituto de Investigación Sanitaria Hospital 12 de Octubre (imas12), Avenida de Andalucía s/n, 28401 Madrid, Spain; 8 Hospital Universitario Puerta de Hierro, Calle Joaquín Rodrigo 1, 28222 Majadahonda, Spain; 9 Hospital General Universitario Gregorio Marañón, Calle Dr. Esquerdo 46, 28007 Madrid, Spain; 10 Philips Healthcare, 28046 Madrid, Spain; 11 School of Medicine, Universidad Complutense de Madrid, Plaza de Ramón y Cajal s/n, 28040 Madrid, Spain

**Keywords:** heart transplant, anti-HLA antibodies, rejection

## Abstract

Introduction: Heart transplant (HT) survival has barely improved in the last decades, which is unsatisfactory for many HT recipients. The development of anti-human leukocyte antigen (anti-HLA) antibodies in HT patients is associated with a cardiac allograft dysfunction. The mechanisms leading to this damage are unclear. The Multimodality Evaluation Of Antibody-Mediated Injury In Heart Transplantation (LEONE-HT) study aimed to thoroughly describe the damage inflicted on the myocardium by anti-HLA antibodies. Methods and analysis: The LEONE-HT study is a cohort study with a cross-sectional approach in which HT patients with positive anti-HLA antibodies are compared with coetaneous HT patients with negative anti-HLA antibodies. All patients will undergo a state-of-the-art multimodal assessment, including imaging techniques, coronary anatomy and physiology evaluations and histological and immunological analyses. The individual and combined primary outcomes of structural graft injuries and longitudinal secondary outcomes are to be compared between the exposed and non-exposed groups with univariate and multivariable descriptive analyses. Ethics and dissemination: The LEONE-HT study is carried out in accordance with the principles set out in the Declaration of Helsinki and the International Conference on Harmonization guidelines for good clinical practice and following national laws and regulations. The study design, objectives and participant centers have been communicated to clinicaltrials.gov (NCT05184426). The LEONE-HT study counts on the support of patient associations to disseminate the objectives and results of the research. This study was funded by the Spanish Ministry of Science and Innovation and the Spanish Society of Cardiology.

## 1. Strengths and Limitations of This Study

- The appearance of anti-HLA antibodies is known to be related to poorer outcomes, suggesting that anti-HLA antibodies induce chronic graft damage beyond acute rejection.
- The LEONE-HT project aimed to describe the structural damage inflicted on the myocardium by anti-HLA antibodies and test the performance of different diagnostic techniques to identify and grade antibody-mediated injuries in HTs.
- Patients underwent a complex multimodal assessment, including state-of-the-art histopathologic, physiologic and imaging techniques.
- Our study limitations were inherent to the observational approach as the selection of non-exposed patients was the most complex part of the study.
- This pilot study aimed to define antibody-mediated damage in HT thoroughly and set the basis for future dedicated studies in the field.

## 2. Introduction

Heart transplantation (HT) is the last therapeutic alternative for many patients with heart failure [1]. In recent years, the short-term survival for HT patients has markedly improved (85–90% survival rate in the first year after HT). However, the long-term survival has barely improved, remaining around 11 years [2]. Although there are many risk factors related to a late allograft dysfunction, the detection of anti-human leukocyte antigen (anti-HLA) antibodies is true for half of the patients [3,4] and the detection of donor-specific antibodies constitutes an independent predictor of a poorer survival in this population [5].

The management of patients with anti-HLA antibodies is not well-defined due to the lack of solid evidence, with no reliable data about “which antibodies are of clinical relevance, which patients to treat, and which treatments are most effective or safe” [6]. Thus, although the treatment of acute antibody-mediated rejection is well-established, there is a lack of evidence for managing chronic antibody-mediated injuries [7]. The current follow-up work-up of HT patients includes echocardiography, coronary angiography and an endomyocardial biopsy. The appearance of de novo anti-HLA antibodies with an otherwise normal work-up is relatively common. However, most of these patients finally develop a graft dysfunction. In this scenario, and for many patients, there are no appropriate treatments and re-transplantation is the only option. Nowadays, there is a trend towards a more aggressive approach to these patients to better understand the pathophysiology of the process. This often leads to the performance of the coronary angiography, right-heart catheterization and endomyocardial biopsy out of the established protocols.

This is why a detailed description of antibody-mediated chronic injuries is a necessary first step to lead us to develop evidence-based diagnostic and care protocols. 

An antibody-mediated injury is produced through the activation of the complement system, which leads to leukocyte migration, increased phagocytosis and a T cell-mediated immune response. The rapamycin pathway promotes cytoskeletal remodeling and immune cell proliferation and migration. This immune response finally leads to allograft vasculopathy and a late allograft failure. The time from the antibody detection to myocardial damage varies between patients from a few months to several years [8].

The vascular wall is an interface between the graft and the immune system of the recipient. The evidence suggests that antibody-mediated injuries are predominantly related to vascular damage [9]. In kidney transplantations, the use of transmission electron microscopy (TEM) offers a detailed description of the antibody-mediated injury where endothelial damage can be identified with endothelial cell enlargement and vacuolization, subendothelial widening and the duplication of the basal membrane. Therefore, the use of TEM has been included in the evaluation of antibody-mediated rejection in kidney transplant patients [10].

Conversely, the diagnosis of antibody-mediated injuries in HT relies solely on optic microscopy (OM) and immunohistochemistry (IHC) techniques [11] because a TEM-based description is unavailable. 

In this study, we aimed to describe the tissue damage inflicted on the myocardium by anti-HLA antibodies in heart transplant patients through a multimodal evaluation, including advanced imaging techniques, tissue and immunological analyses and coronary anatomical and physiological assessments. 

## 3. Methods and Analysis

### 3.1. Study Hypotheses and Objectives

**Figure 1:**
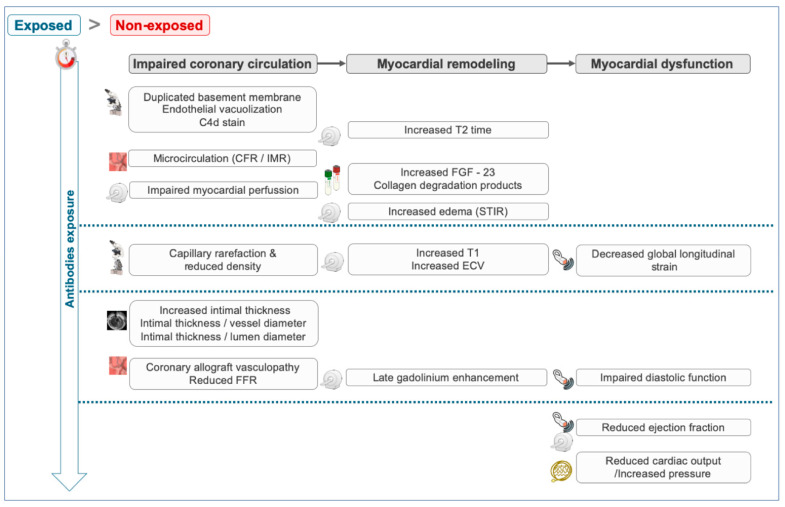
Working hypothesis and expected outcomes. CFR: coronary flow reserve; ECV: extracellular volume; FFR: fractional flow reserve; FGF: fibroblast growth factor; IMR: index of microvascular resistance; STIR: short tau inversion recovery.

Graft injuries related to a chronic exposure to anti-HLA antibodies debuts with microvascular damage and an impaired myocardial perfusion, leading to inflammation, fibrosis and, ultimately, an impaired myocardial function. This microvascular and myocardial damage may be identified early with TEM and with different diagnostic techniques commonly employed in daily clinical practice. The severity of the expected findings will progress depending on the time from the HT and will be more severe in those patients with positive anti-HLA antibodies. Specifically, we expect to find (Figure 1):

- Endothelial cell vacuolization, subendothelial widening and duplicated basal membranes [12].
- An impaired myocardial perfusion evaluated with cardiac magnetic resonance (CMR)-based quantitative perfusion sequences and with invasive parameters (a pressure guidewire) of microvascular damage (coronary flow reserve and an index of microvascular resistance) [13].
- Increased serum and plasmatic markers of fibrosis: fibroblast growth factor 23 (FGF-23), C-terminal propeptide of procollagen type (PICP) and collagen type 1 C-terminal telopeptide (CITP) [14,15].
- Increased T2 recovery times in CMR T2 mapping sequences due to intracellular edemas and increased native T1 recovery times and calculated extracellular volume as a sign of myocardial fibrosis [16].
- Subclinical parameters of a myocardial dysfunction (reduced global longitudinal strain in transthoracic echocardiography) [17].
- Signs of coronary allograft vasculopathy in patients with long-term exposure, including a reduced fractional flow reserve (guidewire pressure) and increased intimal thickness (intravascular ultrasound (IVUS)) [18].
- Diastolic and systolic dysfunctions in patients with an established chronic rejection.

To evaluate these hypotheses, we propose the following study objectives:

- Primary objective: To determine whether, in HT patients, a structural graft injury is modified or accelerated in patients exposed to anti-HLA antibodies compared with non-exposed contemporary HT patients through a thorough evaluation with TEM, OM and IHC techniques.
- Secondary objectives:
  ○ An assessment of the predictive performance of different diagnostic tools (CMR, transthoracic echocardiography, serum markers of fibrosis, intracoronary pressure guidewire and IVUS) to detect tissue damage using TEM, OM and IHC techniques as the combined reference standard.
  ○ An estimation of the risk of adverse outcomes as a function of time from anti-HLA antibody positivity.

### 3.2. Study Population

Our study base is a large dynamic cohort of HT patients assembled in the region of Madrid, Spain. All patients have received an HT and continue their follow-up in one of the three participating centers: University Hospital 12 de Octubre; University Hospital Gregorio Marañón; and University Hospital Puerta de Hierro. The local protocols for the follow-up and treatment of the HT patients are equivalent for the three participating centers. 

**Figure 2:**
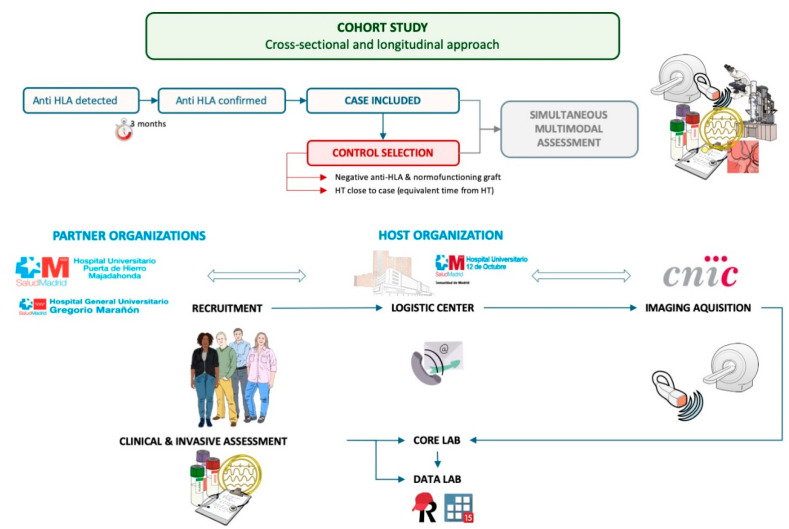
Study design and patient workflow.

For every HT patient in whom anti-HLA antibodies are detected at or since the start of the study (exposed), we will select a comparator (non-exposed) with negative anti-HLA antibodies, a normal functioning graft and whose HT date is close to that of the exposed patient (Figure 2).

A confirmatory test for every patient with positive anti-HLA antibodies will be performed at least three months after the first determination and before the multimodal assessment. A regular anti-HLA antibody determination is performed as part of the local follow-up protocols for HT patients to confirm the patient allocation before evaluating the outcomes for the secondary endpoint. 

All patients will be appropriately informed about the risks of the scheduled test. For the selection of non-exposed patients, we will prioritize those patients who had a routine assessment planned within the local follow-up protocol (especially for the invasive tests).

With the inclusion of a contemporary case of an HT comparator patient (with negative anti-HLA antibodies), we will reduce the possibility of a bias induced by shorter or longer follow-up periods that may under- or over-estimate the effect of anti-HLA antibodies in myocardial injuries. The inclusion and exclusion criteria are defined in Table 1. 

### 3.3. Sample Size Estimation

For our primary objective, we estimated the required sample size based on previous TEM findings for kidney transplants [19]. To find a statistically significant difference in the proportion of patients with established endothelial damage (expected to be 0.29 in the non-exposed group and 0.71 in the exposed group) accepting an alpha risk of 0.05 and a beta risk of 0.2 in a two-sided test, 21 patients were deemed necessary for each group. 

For the secondary objectives, we obtained the estimation based on the previous data of intimal thickness assessments with IVUS: a change of intimal proliferation > 0.5 mm from the baseline to one year after HT was deemed a surrogate marker of allograft vasculopathy [18]. To find a statistically significant proportion difference (expected to be 0.27 in the non-exposed group and 0.6 in the exposed group) with an alpha risk of 0.05 and a beta risk of 0.2 in a two-sided test, we needed a two-sided test to enroll 35 patients for each group.

We expect that, in 10% of the exposed patients, we won’t be able to detect any tissue damage with the current diagnostic tools. Assuming a 10% dropout rate, we plan to recruit 45 patients per group between the 3 participant hospitals.

The recruitment of non-exposed patients may be hampered by difficulties in finding a coetaneous patient for every exposed patient. A contingency plan has been established with an agreement with three additional HT centers in Spain that would start recruiting patients in the case of difficulties in achieving the estimated sample size. 

### 3.4. Study Design

We designed a cross-sectional approach to address the primary objective of this project. 

All exposed and non-exposed participants will undergo a multimodal evaluation that consist of an endomyocardial biopsy and analyses with OM, TEM and IHC as well as transthoracic echocardiography and CMR, right-heart catheterization, coronarography, IVUS, a pressure guidewire assessment, an immunological study and the determination of the serum markers of fibrosis (Figure 2). 

The three participating hospitals are responsible for the patient recruitment and follow-up. The hemodynamic study tissue and blood sampling will be also performed at the recruiting center, according to an *ad hoc* protocol. The Spanish National Center for Cardiovascular Research acts as the imaging core lab, where every included patient is transferred for the imaging acquisition and further analysis. A multidisciplinary team consisting of pathologists, interventional cardiologists, cardiac imaging experts, immunologists and biologists of the host institution (University Hospital 12 de Octubre) will analyzed all performed tests (Figure 2). Those specialist responsible for the analysis are blind to the anti-HLA antibody exposure status of the participants. 

**Figure 3:**
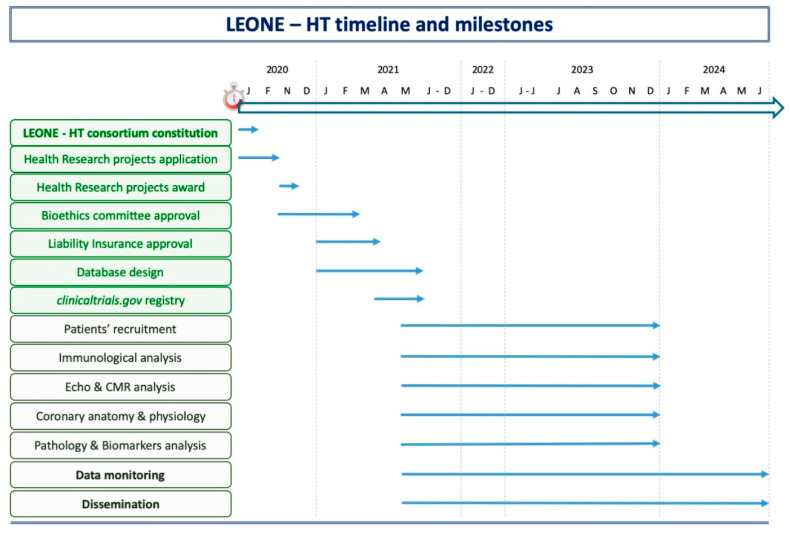
Study timeline and milestones.

Figure 3 summarizes the timeline and the achieved milestones of this research project. 

### 3.5. Assessment Variables

- Primary outcomes: individual and combined structural graft injury outcomes based on the presence of microvascular inflammation, intimal or transmural arteritis or thrombotic microangiopathy (OM); linear C4d staining (%) (IHC); and endothelial edema and vacuolization (semi-quantitative score), subendothelial widening (pm) and basal membrane duplication (TEM).
- For the secondary objectives:
  ○ Microvascular damage assessment: Index of microcirculatory resistance and coronary flow reserve (pressure guidewire), a CMR-based quantitative perfusion assessment (mL/min/100 g of tissue) and the capillary density (capillaries/mm^2^) (OM).
  ○ CMR T2 mapping (ms) to detect intracellular edemas (endothelial vacuolization).
  ○ CMR T1 mapping (ms) and CMR extracellular volume quantification (%) as well as TTE-based global longitudinal strain (%) to identify myocardial remodeling and fibrosis.
  ○ Fibroblast growth factor 23 (R.U./mL), procollagen type 1 carboxy-terminal propeptide (µg/L) and collagen type 1 C-terminal telopeptide (µg/L) as the serum markers of fibrosis and remodeling.
  ○ FFR (coronary physiology) and IVUS-based intimal thickness (mm) as early markers of coronary allograft vasculopathy.
  ○ Rate of antibody-mediated rejection, anti-HLA antibody seroconversion, heart failure and death.

### 3.6. Data Collection and Statistical Analysis 

The study data are collected and managed using REDCap electronic data capture tools hosted at the Instituto de Investigación Sanitaria Hospital 12 de Octubre (imas12). REDCap (Research Electronic Data Capture) is a secure, web-based software platform designed to support data capture for research studies [20,21]. 

A clinical research coordinator (CRC) conducts the data management at the host institution. The CRC is responsible for reviewing the eCRFs and ensuring compliance with the registration protocol. Of the sample, 10% will be randomly selected for the data verification. 

The individual and combined primary outcomes of the structural graft injury and longitudinal secondary outcomes will be compared between the exposed and non-exposed groups with univariate and multivariable descriptive analyses. The predictive performance of CMR, TTE, coronary physiology parameters and IVUS will be assessed with univariate and multivariable modelling. A parsimonious approach will be implemented to propose a standard assessment protocol, including a predictive score based on the study results. All statistical analyses will be obtained using dedicated software (StataCorp. 2021. Stata Statistical Software: Release 17. StataCorp LLC., College Station, TX, USA).

### 3.7. Patients and Public Involvement

Despite an improvement in the short-term survival of HT patients, the current life expectancy remains unsatisfactory for most of them. The development of anti-HLA antibodies is associated with a worse survival. As the mechanisms leading to these poorer outcomes are unknown, delving into them can help to improve the quality of life and survival of HT patients. 

For the dissemination of this study, we count on the support of several patient associations. HT patients are especially aware of the importance of research in healthcare and we regularly collaborate with patient associations such as the Menudos Corazones Foundation (https://www.menudoscorazones.org, accessed on 20 September 2022), the Hospital 12 de Octubre Association of Transplanted Patients and the Corazón y vida Foundation (https://www.corazonyvida.org, accessed on 20 September 2022). 

## 4. Ethics and Dissemination

### 4.1. Ethical Considerations

This study is carried out in accordance with the principles set out in the Declaration of Helsinki and the International Conference on Harmonization guidelines for good clinical practice and following national laws and regulations. The Ethics Committee approved the study for the clinical research of all participating centers (CEIm 20/518). 

The local investigator of each participating center will inform the patient about all pertinent aspects of the study, including the favorable written report of the Ethics Committee for Clinical Research. 

The ethics committee will be also informed of any event that may have affected patient safety or the continuation of the study.

### 4.2. Dissemination

The design of this study and the participating institutions were declared to the United States National Library of Medicine (clinicaltrials.gov) (NCT05184426).

The results obtained from this research project will continue to be communicated to the scientific community through the participation of the researchers in national and international conferences. Other publications regarding the main results are expected, as are several publications regarding the technical particularities of the diagnostic techniques employed.

## 5. Conclusions

Developing anti-HLA antibodies is associated with a worse prognosis in heart transplant recipients. The mechanism by which these antibodies lead to a graft dysfunction has not been clearly defined. The LEONE-HT study aims to carry out a detailed description of the damage that these antibodies inflict on the transplanted heart to improve knowledge about this phenomenon and thus be able to contribute to the development of diagnostic protocols and the description of possible therapeutic targets.

## Figures and Tables

**Table 1:** Inclusion and exclusion criteria.

Inclusion Criteria	Exclusion Criteria
**For Exposed Patients**	Recipient of a second HT Multiple organ transplantation Unknown immunological history Recipients sensitized with anti-HLA antibodies against donor HLA before HT CMR contrast not administered with GFR < 30 mL/min/1.73 m^2^ Patients with implantable cardiac devices who did not undergo a CMR study
- Recipient of a first HT - De novo anti-HLA detection: Mean fluorescence intensity > 2000 (DSA) Standard fluorescence intensity > 150,000 (NDSA) - Detailed immunological history: Anti-HLA assessment before HT Serial assessment during HT follow-up - Known HLA typing of donor	
**For Non-Exposed Patients**	
Recipient of a first HT Negative anti-HLA antibodies HT contemporary to the index case	

CMR: cardiac magnetic resonance; DSA: donor-specific antibodies; GFR: glomerular filtration rate; HLA: human leukocyte antigen; HT: heart transplantation; NDSA: non-donor-specific antibodies.

## Data Availability

Not applicable.
